# Myelofibrosis and acquired hemophilia A: a case report

**DOI:** 10.1186/s13256-016-0897-4

**Published:** 2016-05-07

**Authors:** Marie Wrobel, Emilie Comio, Valerie Gay, Noureddine Baroudi, Pascal Meyer, Christine Chuniaud-Louche, Maya Hacini, Gian Matteo Pica

**Affiliations:** 1 Service of Hematology, Centre Hospitalier Metropole Savoie, 73011 Chambéry, France; 2 Medical laboratory, Centre Hospitalier Metropole Savoie, 73011 Chambéry, France; 3 Haemophilia center and Transfusion medicine, Centre Hospitalier Metropole Savoie, 73011 Chambéry, France; 4 Hospital Pharmacy, Centre Hospitalier Metropole Savoie, 73011 Chambéry, France

**Keywords:** Acquired hemophilia A, Myelofibrosis, Rituximab, Azacytidine, Activated prothrombin complex concentrates

## Abstract

**Background:**

Myelofibrosis and acquired hemophilia A is a rare association. To the best of our knowledge only one case of myelofibrosis and acquired hemophilia A has been previously described.

**Case presentation:**

A 66-year-old Caucasian man diagnosed with myelofibrosis evolving in acute myeloid leukemia was referred to us for postoperative bleeding. Hemostatic studies showed prolonged activated partial thromboplastin time, decreased factor VIII coagulation, and a high factor VIII inhibitor titer; these findings led to a diagnosis of acquired hemophilia A for which he was treated with methylprednisolone and recombinant activated factor VII on admission. Due to a lack of response he was subsequently treated with rituximab combined with activated prothrombin complex concentrates. Furthermore, he received azacytidine to treat the underlying hematological malignancies. Immunosuppressive rituximab therapy resolved acquired hemophilia A with marked efficacy.

**Conclusions:**

Rapid and accurate diagnosis, effective hemostatic therapy, and timely treatment for underlying disease are important in the management of acquired hemophilia A secondary to hematological malignancy.

## Background

Hemophilia A, also called factor VIII (FVIII) deficiency or classic hemophilia, is a genetic disorder caused by missing or defective FVIII, a clotting protein. Acquired hemophilia A (AHA) is a bleeding disorder caused by autoantibodies against FVIII with an estimated incidence of 1.48 cases per million people per year [1]. Although the underlying disorder cannot be identified in 50 % of patients, it is believed that AHA is associated with autoimmune disorders, malignancies, pregnancy, dermatological disorders, and drug reactions [2]. Surgery has been recognized as a possible etiologic factor or, at least, a possible precipitating cause [3]. The first sign of AHA is usually sudden, massive, life-threatening bleeding occurring in a patient without previous coagulation disorders.

Myelofibrosis (MF) is a clonal proliferation of a pluripotent hematopoietic stem cell [4] in which the abnormal cell population releases several cytokines and growth factors in the bone marrow. Those lead not only to a marrow fibrosis and stromal changes, but also to a pathological cellular homing and trafficking, explaining the so diffusely observed extramedullary hematopoiesis in MF [5].

We describe a case of AHA in a man with MF; it is the second case of MF associated with AHA to be reported in the literature [6] which makes the findings rare and valuable.

## Case presentation

Our patient, a 66-year-old Caucasian man, was diagnosed with MF 4 years ago. He was a high-risk patient according to the Dynamic International Prognostic Scoring System-plus classification because of a platelet (PLT) count of <100 giga/L, transfusion-dependency, hemoglobin (Hb) <100 g/L, circulating blood blasts >1 %, complex karyotype, leucocyte count <25 giga/L, and no constitutional symptoms [7]. His medical history was unremarkable except for peripheral artery disease treated with acetylsalicylic acid. He had neither a personal nor a family history of spontaneous bleeding.

At diagnosis, for MF-related anemia, we started a therapy with recombinant human erythropoietin that was stopped after 3 months for lack of efficacy. He was not considered fit enough for bone marrow transplantation by a specialized center because of his age and a lack of a suitable donor. He received regular red blood cell transfusions, without chelation therapy. Ruxolitinib was contraindicated due to thrombocytopenia.

In order to ameliorate the anemia an indication for splenectomy was given and performed by our regional referral center. The preoperative tests showed a prolonged activated partial thromboplastin time (APTT; 43.8 seconds, normal range 26.8 to 42 seconds). No further investigations were done at that time. Immediately after the intervention he experienced hemorrhagic shock due to bleeding of his phrenic artery which needed a second surgery for hemostasis.

He presented significant abdominal pain 35 days after splenectomy that led to a CT scan which showed a hematoma in his pancreatic loge. We referred him to our regional center for a percutaneous drainage. He was finally readmitted to our Department for the postoperatory phase. Repeated laboratory tests found a gradual elongation of APTT: 52 seconds on day 51 and 114 seconds on day 61 from splenectomy (normal range, 26.8 to 42 seconds). A physical examination detected a large ecchymosis on his left arm. The same day we completed coagulation tests that showed a deficiency in FVIII coagulant activity (FVIII: C): <1 % (normal range, 50 to 150 %). Prothrombin time (PT), von Willebrand factor and other clotting factors (II, V, VII, IX, X, and XI) were all normal. FVIII inhibitor (AAFVIII) was positive at a titer of 17.3 Bethesda units (BU). He was diagnosed as AHA based on the above findings (day 0).

Moreover, hematological examinations showed leukocytosis (white blood cell count 24×10^9^/L) with marked leukoerythroblastosis (blasts cells 25 %), Hb of 113 g/L, and thrombocytopenia (PLT count 57×10^9^/L). These findings were consistent with the diagnosis of leukemic transformation according to the World Health Organization (WHO) criteria [8]. A bone marrow examination was not performed due to the excessive bleeding risk.

Immunosuppressive therapy with methylprednisolone 1 mg/kg body weight was promptly started. Due to the apparition of deep muscle hematomas of his arms and legs we added recombinant activated factor VII (rFVIII; NovoSeven®, Novo Nordisk) 90 mcg/kg every 2 hours. On day 4 from AHA diagnosis we associated rituximab (MabThera®) 375 mg/m^2^ weekly for 4 weeks to the ongoing steroid therapy. Due to a lack of response, on day 6 we stopped the rFVIII and started activated prothrombin complex concentrate (aPCC; FEIBA®) intravenously at a dose of 80 unit/kg every 12 hours. aPCC was subsequently decreased to 80 unit/kg every 24 hours on day 10, then stopped at day 18.

To treat the leukemic transformation of the MF that probably was associated to the AHA, on the sixth day our patient started chemotherapy with azacytidine (5-AZA) administered subcutaneously at 75 mg/m^2^/day for 7 days [9]. Red blood cell and PLT transfusion were also carried out as supportive therapy. After 10 days of treatment his edema and ecchymosis showed a marked improvement. The AAFVIII rose to 179 BU on day 12 and descended to 25 BU on day 33.

During the post-chemotherapy phase he presented *Staphylococcus hominis* bacteremia treated with intravenous vancomycin. Subsequently he was healed for a probable *Pneumocystis* pneumonia by co-trimoxazole and atovaquone. He was referred to our out-patient care department on day 40.

**Fig. 1:**
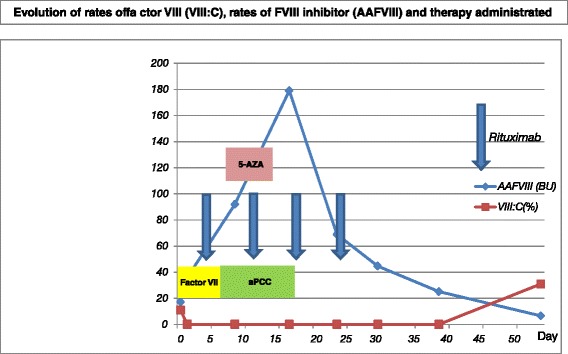
This graph shows factor VIII inhibitor activity and factor VIII coagulant activity levels. The patient was treated with recombinant activated factor VII and activated prothrombin complex concentrate for 18 days. He was also given rituximab weekly for four administrations and one cycle of azacytidine, then factor VIII inhibitor disappeared and factor VIII coagulant activity gradually returned to normal. *5-AZA* azacytidine, *AAFVIII* factor VIII inhibitor, *aPCC* activated prothrombin complex concentrate, *VIII: C* factor VIII coagulant activity

On day 57, before his second cycle of 5-AZA, his FVIII: C was elevated to 30 % with no additional bleeding episodes; his APTT was decreased to 72 seconds. AAFVIII was decreased to a titer of 6.6 BU. Evolution of rates of FVIII (VIII: C), rates of AAFVIII, and therapy administered are summarized in Fig. 1.

## Discussion

AHA is diagnosed in patients without a personal or family history of bleeding, who have prolonged APTT, reduced FVIII levels, and detectable specific AAFVIIIs. Surgery is known to be a precipitating cause of acquired hemophilia, which is something to be considered with unusual postoperative bleeding [10, 11]. In our case, due to the intervening period of approximately 2 months, we did not consider surgery to be directly associated with the inhibitor, although it might have played a role in its development. While it is believed that AHA is caused by autoantibodies against FVIII, the pathogenesis of AHA is still unclear. One possible mechanism is the production of autoantibodies as a result of the breakdown of peripheral immune tolerance mechanisms that regulate immune responses to FVIII. Moreover, it has been reported that FVIII-specific CD4+ T cells are crucial for the development of AHA [12]. According to the literature, underlying malignancies are present in 10 % of AHA cases [13]. The malignant clone of tumor cells has a negative regulatory role in recognizing foreign antigens of CD4+ cells. Immune dysfunction in patients with a tumor may result in the development of AAFVIIIs. Alternatively, it is possible that FVIII autoantibodies are triggered by cancer cells or cancer cell products, which possibly contain antigen material similar to FVIII [14].

The association between AHA and hematologic malignancies, especially lymphoproliferative disorders, is in line with the broad range of autoimmune phenomena that frequently complicate these conditions [15]. Nevertheless, association between AHA and myeloid hematological malignancies has been reported [16, 17]. In MF, clonal myeloproliferation is associated with reactive bone marrow fibrosis, osteosclerosis, angiogenesis, extramedullary hematopoiesis, and abnormal cytokine expression that may explicate the development of an autoimmune disorder [18]. One case of this rare association has been previously described [6]. The principle of AHA treatment is to control the severe hemorrhage, eradicate the inhibitors by immunosuppressive therapy, and treat underlying disorders. It is recommended to immediately initiate anti-hemorrhagic treatment and eradication of autoantibodies in patients with AHA who present with active severe hemorrhage, and to avoid invasive procedures. Currently, recombinant factor VIIa and aPCC (FEIBA®) are the available bypassing agents in the USA and Europe.

## Conclusions

MF associated with AHA is a challenge to diagnose, considering that AHA is still not a widely known condition. AHA should be suspected immediately in patients who have a malignant disease, bleeding, and isolated prolongation value of APTT. Surgery could be a precipitating cause of acquired hemophilia. This syndrome is remarkable for its abrupt onset within days of surgery, dramatic bleeding, subsequent persistence, but potential reversal by immunosuppression. Identifying and treating the underlying disease in AHA cases might help to control this fatal condition.

In our case, clinical remission of AHA was achieved with the combination of hemostatic therapy, immunosuppressive treatment (steroids and rituximab), and anti-leukemia therapy with the epigenetic drug 5-AZA, which shows the importance of treating the underlying disease in AHA cases. The findings in this case indicate that rapid and accurate diagnosis, effective hemostatic therapy, and timely treatment for underlying disease are important in the management of AHA.

## Consent

Written informed consent was obtained from the patient for publication of this case report and any accompanying images. A copy of the written consent is available for review by the Editor-in-Chief of this journal.

## Abbreviations

5-AZA: azacytidine
AAFVIII: factor VIII inhibitor
AHA: acquired hemophilia A
aPCC: activated prothrombin complex concentrate
APTT: activated partial thromboplastin time
BU: Bethesda units
FVIII: factor VIII
FVIII: C: factor VIII coagulant activity
Hb: hemoglobin
MF: myelofibrosis
PLT: platelet
PT: prothrombin time
rFVII: recombinant activated factor VII
WHO: World Health Organization
